# Evolution of adrenoleukodystrophy model systems

**DOI:** 10.1002/jimd.12357

**Published:** 2021-01-07

**Authors:** Roberto Montoro, Vivi M. Heine, Stephan Kemp, Marc Engelen

**Affiliations:** ^1^ Department of Pediatric Neurology, Emma Children's Hospital, Amsterdam UMC, Amsterdam Leukodystrophy Center, Amsterdam Neuroscience University of Amsterdam Amsterdam The Netherlands; ^2^ Department of Child and Youth Psychiatry, Amsterdam UMC, Amsterdam Neuroscience Vrije Universiteit Amsterdam Amsterdam The Netherlands; ^3^ Department of Complex Trait Genetics, Centre for Neurogenomics and Cognitive Research, Amsterdam Neuroscience Vrije Universiteit Amsterdam Amsterdam The Netherlands; ^4^ Department of Clinical Chemistry, Laboratory Genetic Metabolic Diseases, Amsterdam UMC, Amsterdam Gastroenterology & Metabolism University of Amsterdam Amsterdam The Netherlands

**Keywords:** fatty acids, inborn error of metabolism, model systems, pathogenesis, peroxisomes

## Abstract

X‐linked adrenoleukodystrophy (ALD) is a neurometabolic disorder affecting the adrenal glands, testes, spinal cord and brain. The disease is caused by mutations in the *ABCD1* gene resulting in a defect in peroxisomal degradation of very long‐chain fatty acids and their accumulation in plasma and tissues. Males with ALD have a near 100% life‐time risk to develop myelopathy. The life‐time prevalence to develop progressive cerebral white matter lesions (known as cerebral ALD) is about 60%. Adrenal insufficiency occurs in about 80% of male patients. In adulthood, 80% of women with ALD also develop myelopathy, but adrenal insufficiency or cerebral ALD are very rare. The complex clinical presentation and the absence of a genotype‐phenotype correlation are complicating our understanding of the disease. In an attempt to understand the pathophysiology of ALD various model systems have been developed. While these model systems share the basic genetics and biochemistry of ALD they fail to fully recapitulate the complex neurodegenerative etiology of ALD. Each model system recapitulates certain aspects of the disorder. This exposes the complexity of ALD and therefore the challenge to create a comprehensive model system to fully understand ALD. In this review, we provide an overview of the different ALD modeling strategies from single‐celled to multicellular organisms and from in vitro to in vivo approaches, and introduce how emerging iPSC‐derived technologies could improve the understanding of this highly complex disorder.

## Graphical abstract

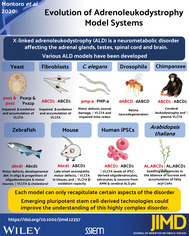

**SYNOPSIS**

This review describes the advantages and limitations of currently available ALD models and anticipates the impact of iPSC technology on future models.

## 1 MODEL SYSTEMS ARE ESSENTIAL FOR PRECLINICAL RESEARCH

X‐linked adrenoleukodystrophy (ALD; OMIM: 300100) is the most common peroxisomal neurometabolic disorder characterized by a spectrum of symptoms and defined by mutations in the *ABCD1* gene.^1^ Nearly all men and ~80% of women develop slowly progressive spinal cord disease known as adrenomyeloneuropathy (AMN). In men, features range from adrenal insufficiency to progressive inflammatory cerebral demyelination (cerebral ALD), which are very rare findings among women with ALD.^1^, ^2^ Moreover, given that monozygotic twins can have a disconcordant disease course, a combination of rare genetic modifiers, epigenetic, and environmental factors have been hypothesized to impact the disease outcome.^3^, ^4^ The complex clinical presentation and the absence of a genotype‐phenotype correlation are complicating our understanding of the disease.

Model systems have been extensively employed in an attempt to understand the pathophysiology of ALD (Table 1). Truncation of the *ABCD1* gene has been the most used strategy to experimentally model ALD. in vitro studies provided us the majority of knowledge on ABCD1 biochemistry and function, but obviously come with limitations when trying to understand a neurological dysfunction. In addition, the creation of animal knockout models of the human *ABCD1* ortholog has advanced our insights into disease mechanisms. However, animal models also failed to fully recapitulate the complex neurodegenerative etiology of ALD. Recently, the ALD field has begun to explore the potentials of induced pluripotent stem cell (iPSC)‐based modeling approaches. The first patient iPSC studies presented biochemical hallmarks of ALD in disease‐relevant cell types of the nervous system.^5^, ^6^

**TABLE 1:** A summary of the main findings of the model systems used to study ALD

Model system	Gene	Protein	Main findings
Fibroblasts	*ABCD1*	ABCD1	Impaired β‐oxidation and accumulation of VLCFA
Yeast	*pxa1* and *pxa2*	Pxa1p and Pxa2p	Impaired β‐oxidation and accumulation of VLCFA
*C. elegans*	*pmp‐4*	PMP‐4	Motor defects, axonal damage, VLCFA accumulation, and impaired mitochondrial redox
Drosophila	*dABCD*	dABCD	Retina neurodegeneration
Zebrafish	abcd1	Abcd1	Motor defects, developmental deficiencies in *olig2* ^+^ progenitors of oligodendrocyte and motor neuron, VLCFA, and cholesterol accumulation
Mouse	*Abcd1*	ABCD1	Late onset axonopathy, motor defects, elevated levels of VLCFA in tissues, and decreased VLCFA β‐oxidation capacity
Chimpanzee	*ABCD1*	ABCD1	Cerebral leukodystrophy and elevated VLCFA plasma levels
Human iPSCs	*ABCD1*	ABCD1	Elevated VLCFA levels of iPSC‐derived oligodendrocytes, astrocytes, and neurons from AMN and cerebral ALD patients
*Arabidopsis thaliana*	*At_ABCD1*	At_ABCD1	Seedling deficiencies in the absence of sucrose and accumulation of fatty acyl CoAs

Each model system recapitulates certain aspects of the disorder. This exposes the complexity of ALD and therefore the challenge to create a comprehensive model system to fully understand ALD. In this review, we provide an overview of the different ALD modeling strategies from single‐celled to multicellular organisms and from in vitro to in vivo approaches, and introduce how emerging iPSC‐derived technologies could improve the understanding of this disorder.

## 2 BIOCHEMICAL INSIGHTS ON ABCD1 FUNCTION FROM UNICELLULAR MODELS

Elevated levels of very long‐chain fatty acids (VLCFA, ≥C22:0) in plasma and tissues represent the biochemical signature of ALD.^7^ Elevated levels of VLCFA were first demonstrated in fibroblasts from ALD patients. The postulation that a defect in VLCFA metabolism is central to the pathogenesis of ALD was confirmed by the demonstration that fibroblasts from ALD patients have a reduced capacity to degrade VLCFA.^8^ In addition, the finding that degradation of C16:0, which is a substrate for mitochondrial β‐oxidation, was completely normal in ALD cells suggested that VLCFA breakdown involves a specific metabolic pathway that is distinct from long‐chain fatty acids (LCFAs).^9^ This notion was supported by the findings that VLCFA β‐oxidation was also deficient in fibroblasts of patients with the peroxisome biogenesis disorder Zellweger syndrome.^10^ The final demonstration that ALD is a peroxisomal disease came from the identification of pathogenic mutations in the *ABCD1* gene.^11^ Indeed, retroviral‐mediated transfer of the ABCD1 cDNA in ALD fibroblasts corrected VLCFA β‐oxidation to normal levels.^12^ Thus, ALD is a peroxisomal disorder identified by mutations in *ABCD1*, which encodes for the peroxisomal ATP‐binding cassette sub‐family D member 1 (ABCD1), and is biochemically characterized by high VLCFA levels.

Initial insights into ABCD1 function came from studies in yeast. Deletions in the yeast orthologs of the human ABCD transporters, that is, Pxa1p and Pxa2p, disrupted the import of LCFAs into peroxisomes, and subsequently their β‐oxidation.^13^ Owing to the high similarity between ABCD1 and the yeast ABC transporters, together with the common biochemical deficiencies in the β‐oxidation of fatty acids, Hettema and colleagues suggested that ABCD1 might be involved in the uptake of VLCFAs into the human peroxisomes.^13^ In agreement with these findings, expression of human ABCD1 in the *pxa1/pxa2*Δ double mutant partially restored decreased β‐oxidation activity levels. This provided the first evidence that homodimeric ABCD1 may indeed transports VLCFA into the peroxisomes. Due to technical challenges, it still took >15 years before it was demonstrated that ABCD1 transports VLCFA, as their CoA esters, across the peroxisomal membrane.^14^ Using purified peroxisomes from ALD patient fibroblasts, Wiesinger et al demonstrated that β‐oxidation of VLCFacyl‐CoA esters directly depends on ABCD1.^15^ Together, these findings confirmed the function of ABCD1 in VLCFA metabolism and established that ABCD1 is an integral peroxisomal membrane transporter that shuttles VLCFA CoA‐esters into the peroxisome for β‐oxidation.

In addition to ABCD1, the peroxisomal membrane harbors two additional ABC transporters: the adrenoleukodystrophy‐related protein (ALDPR/ABCD2)^16^ and the 70 kDa peroxisomal membrane protein (PMP70/ABCD3).^17^ Yeast *pxa1/pxa2*Δ proved to be useful in identifying the substrate that is specific for the human ABCD transporters. The saturated fatty acids C24:0 and C26:0 are preferentially imported by ABCD1,^18^ while polyunsaturated fatty acids are transported by ABCD2,^18^ and dicarboxylic acids by ABCD3.^19^ Interestingly, overexpression studies showed that ABCD1, ABCD2, and ABCD3 can have overlapping substrate functions, although each transporter presents distinctive substrate preferences under normal physiological conditions.^18^, ^19^ Substrate specificity also plays a role in the case of the fatty acids elongases. Expression of the seven human ELOVL enzymes in yeast revealed ELOVL1 as the single elongase that catalyzes the synthesis of both saturated VLCFA (C26:0) and monounsaturated VLCFA (C26:1) in humans.^20^ Expression of ELOVL1 is not increased in ALD patient fibroblasts, indicating that elongation of VLCFAs is increased in ALD patients due to elevated substrate availability.^20^, ^21^ Moreover, deuterium tracing of newly synthesized VLCFA in ALD fibroblast cultures demonstrated that the elongated fatty acids are incorporated into complex lipids.^21^ Therefore, the primary deficiency of ABCD1 results in accumulated cytosolic VLCFA‐CoA levels, which are substrate for further chain lengthening by ELOVL1. These fatty acids are incorporated into complex lipids.

The peroxisomal ABCD proteins are half‐transporters that have to dimerize to form a functional unit. Early studies using the yeast two‐hybrid system revealed that the carboxyl‐terminal halves of ABCD1‐3 transporters can homo‐ as well as heterodimerize.^22^ By co‐immunoprecipitation and FRET analyses, the presence of both homo‐ and heterodimeric structures was confirmed.^23^, ^24^ Functionality of homodimers is further supported as expression of each ABCD1‐3 alone restored β‐oxidation levels in *pxa1/pxa2*Δ mutants.^14^, ^19^ Chimeric expression of homo‐ and heterodimers in *pxa1/pxa2*Δ yeast mutants showed that both are functional conformations.^25^ Moreover, their expression in primary fibroblasts from ALD patients partially reduced VLCFA levels.^25^ Noteworthy, a recent characterization of the quaternary structure revealed the existence of ABCD1 and ABCD2 as tetramers in the peroxisomal membrane, mainly as homotetramers.^26^ However, the functional significance of these heterodimers remains unknown. Overall, the peroxisomal transporter ABCD1 has been shown to preferentially function as a homodimer in vivo. Whether the heterodimers that are formed in vitro also occur in vivo has yet to be proven.

## 3 MULTICELLULAR ORGANISMS MODELING OF ALD

A great diversity of animal models have been generated in an effort to understand how deficiencies in peroxisomal transport of fatty acids and subsequent accumulation contribute to the axonal loss and demyelination seen in ALD patients.

## 4 MITOCHONDRIAL COMPENSATION IN *CAENORHABDITIS ELEGANS*

Recently, a nematode model of ALD has been developed by mutating the peroxisomal membrane protein *pmp‐4*, which is the *Caenorhabditis elegans* ortholog of the mammalian ABCD transporters ABCD1 and ABCD2.^27^ Deficiencies in PMP‐4 lead to VLCFA accumulation, impaired mitochondrial redox homeostasis, and motor behavior defects associated with axonal damage.^27^ In line with other peroxisomal mutants, *pmp‐4* worms presented increased total numbers of lipid droplets with a diameter >5 μm. During starvation the main fuel comes from lipid degradation. However, fasted *pmp‐4* worms were unable to make use of their lipid reserves. Intriguingly, mitochondrial antioxidant treatment normalized the lipid droplet amount and size. This scenario uncovers the possibility that mitochondria may compensate peroxisomal impairment and degrade lipid droplet‐derived fatty acids. Additional studies are needed to reveal the impact on axonal degeneration by the proposed axis between the loss of peroxisomal function, lipid droplet accumulation, and mitochondrial redox imbalance.

## 5 RETINA NEURODEGENERATION IN DROSOPHILA

A variety of drosophila models have been developed to study VLCFA metabolism. Disruption of the drosophila orthologue of the human *ABCD1*, termed *dABCD*, results in an age‐dependent neurodegeneration specified by retinal holes and pigment cell loss.^28^ Interestingly, cell type‐specific knockdown of *dABCD* revealed that retinal defects are only seen when neuronal, but not glial *dABCD* is targeted.^28^ This warrants further characterization of the *dABCD1* model.

Free fatty acids are, in general, metabolically inactive. To be transported by ABCD1 into the peroxisome, VLCFAs have to be thioesterified to coenzyme A (VLCFA‐CoA) by acyl‐CoA synthetases.^29^ *Bubblegum* (*bgm)* and *double bubble* (*dbb)* have been identified as the long‐ and very‐long‐chain acyl‐CoA synthetases in Drosophila.^30^, ^31^ Increased VLCFA levels are present in *bgm/dbb* double mutant flies, but not in the single mutants. *Bgm* and *dbb* single mutants show laminal and retinal degeneration, thinning, and irregularity of the fenestrated membrane, and decreased locomotion. A diet rich in the medium‐chain fatty acids significantly reduced the retinal defects in *bgm* and *dbb* mutants.^28^ Meanwhile, supplementation by LCFAs did not worsen the phenotype, which supports the idea proposed by Gordon et al^28^ that neurodegeneration results from lack of VLCFA metabolic products.

Once inside the peroxisome, acyl‐CoA oxidase 1 (ACOX1) is the rate‐limiting enzyme for the β‐oxidation of VLCFA‐CoA esters.^32^ In drosophila, *dACOX1* is highly expressed in glia, including perineural glia and wrapping glia, while poorly expressed in neurons.^33^ Loss of *dACOX1* leads to lifespan reduction, increased VLCFA levels, retina degeneration, reduced motor skills, and failure of wrapping glia to ensheath axons that consequently leads to axonal loss. Pharmacological treatment of *dACOX1*‐deficient flies with bezafibrate, which directly inhibits C26:0 synthesis through a direct inhibition of ELOVL1,^34^, ^35^ suppressed lethality, improved locomotion, and ameliorated the synaptic transmission and integration in the retina. Surprisingly, a much higher dose was needed to reduce C26:0 levels in fibroblasts compared to the dose required to observe an effect in flies.^34^ Accordingly, knockdown of *dELOVL1* by RNAi led to the same phenotype improvements.^33^ Together, these findings provide strong support for the hypothesis that elevated levels of VLCFAs in glia promotes neurodegeneration.

On the other hand, *dACOX1* gain‐of‐function in larvae results in increased levels of reactive oxygen species (ROS) and lethality, which can be overcome by treatment with antioxidant, *N*‐acetyl cysteine amide (NACA).^33^ Tissue‐specific *dACOX1* gain‐of‐function determined that the glial‐ and wrapping glia‐specific drivers impact viability most. Also, *dACOX1* gain‐of‐function in wrapping glia causes locomotion defects that can be rescued by treatment with catalase, including normalization of ROS levels. In rat, overexpression of *ACOX1* and its gain‐of‐function caused Schwann cell death, which is reversed by treatment with NACA.^33^ ACOX1 was shown to be present in myelinating Schwann cells but not in the axons they ensheath. Noteworthy, a patient carrying the *ACOX1* gain‐of‐function variant showed severe demyelination, loss of Schwann cells, and neurodegeneration.^33^

These insights in *dABCD1* led to the following questions: Do *dABCD1* mutants also accumulate VLCFAs? What is the expression pattern of *dABCD1*? How is wrapping glia affected in *dABCD1*mutants?

## 6 A DEVELOPMENTAL VIEW OF ALD FROM ZEBRAFISH

Zebrafish *abcd1* mutants present severely disrupted development of the CNS and the interrenal organ, the zebrafish homolog of the mammalian adrenal glands. Importantly, *abcd1* mutants display elevated VLCFA levels, cholesterol accumulation, and motor impairments.^36^, ^37^ Gene expression analysis revealed that *olig2* ^*+*^ oligodendrocyte progenitor cells (OPCs) in the developing brain and spinal cord express *abcd1*. Accordingly, *abcd1*‐deficient OPCs have a profound effect on myelin development and oligodendrocyte generation, as shown by reduced expression of the myelin protein proteolipid protein 1a (*plp1a*), a decreased percentage of myelinated axons in the spinal cord, and an incorrect patterning of OPCs.^37^ Although there was an increase in apoptosis in the mutant brains, programmed cell death was not specific to OPCs. Interestingly, expression of human *ABCD1* under *sox10* promoter reduced apoptosis, rescued the deficient numbers and affected patterning of OPCs, and improved locomotion behavior. Given that in addition to oligodendrocytes, *olig2* ^*+*^ progenitors also give rise to motor neurons,^38^ follow up studies on this ALD model could determine the impact of abcd1 defects on motor neuron development and function.

These findings drawn from zebrafish suggest that there might be an underlying developmental component of the neurodegenerative disorder. In a developmental view of ALD depicted by zebrafish modeling, deficiencies in the progenitor pool of oligodendrocytes and motor neurons in early development would lead to an increased susceptibility to develop cerebral demyelination or neurodegeneration later in life.

## 7 MOUSE MODELS PRESENT NEURONAL AND GLIAL INVOLVEMENT

In 1997, three independent laboratories reported the generation of an ALD mouse model.^39^, ^40^, ^41^ All three models were generated by gene targeting which completely abolished ABCD1 protein expression. The ALD mouse recapitulates the key biochemical features of ALD: fibroblasts generated from Abcd1 null mice have decreased VLCFA β‐oxidation capacity^41^ and tissues have elevated levels of VLCFAs^39^, ^40^, ^41^, ^42^ and lipid inclusions in adrenals, but not in the CNS.^39^ Despite these key features, ALD mice do not develop adrenal insufficiency^43^ or cerebral ALD. Instead, ALD mice develop a late‐onset axonopathy and locomotor impairment at 20 months of age.^44^ ALD mice develop an isolated spinal cord phenotype that resembles the late onset seen in women with ALD.^2^, ^45^ Already at 3.5 months of age, motor neurons of the spinal cord display oxidative stress as well as oxidative, glycoxidative, and lipoxidative damage to proteins.^42^ Excess amounts of VLCFA impair mitochondrial oxidative phosphorylation (OXPHOS) and key enzymes of the tricarboxylic acid (TCA) cycle in the spinal cord of *Abcd1* null mice, but not in other tissues.^46^, ^47^, ^48^ This cascade of redox imbalances originates from the combined effect of increased mitochondrial ROS caused by the increased levels of VLCFA,^49^ along with impairment of the proteasome, autophagy, and antioxidant systems,^50^, ^51^, ^52^, ^53^ which ultimately leads to axonal degeneration in the *Abcd1* null mouse. In the ALD mouse, oxidative stress is associated with axonal degeneration as a combination of the antioxidants α‐tocopherol, *N*‐acetylcysteine, and α‐lipoic acid ceased axonal damage, reversed locomotor capabilities, and reduced elevated ROS.^49^ Furthermore, this treatment prevented energetic dysfunction^46^ and rescued deficiencies in the protein homeostasis network.^51^, ^52^ In a recent small phase II pilot open‐label study, 13 patients with AMN received a combination of high‐dose α‐tocopherol, *N*‐acetylcysteine, and α‐lipoic acid. The primary outcome of the study was the validation of a panel of oxidative damage and inflammation biomarkers^54^ which normalized upon treatment. In addition, an improvement on the 6‐minutes walk test was reported in some patients justifying a larger placebo‐controlled trial in the future.

To model severe neurological manifestations of ALD, *Abcd1* null mice have been crossed to several other mice carrying mutations that are considered possible modifier genes for ALD (Table 2). Compared to single mutants, double *Abcd1/Abcd2* KO mice show a more severe AMN phenotype accompanied by higher VLCFA accumulation in the spinal cord and adrenal glands,^55^ locomotion impairment at an earlier onset, greater levels of oxidative damage in the spinal cord,^56^ and presence of inflammatory infiltrates of T lymphocytes in the spinal cord.^55^ In another effort to obtain a more relevant clinical phenotype, ELOVL1 was overexpressed in Abcd1^y/−^ mice.^57^ While ELOVL1 overexpression revealed that oligodendrocytes account for the majority of VLCFA synthesis and degradation in brain, the *Abcd1* ^*−/y*^ */ELOVL1* ^*+/−*^ mice did not worsen the phenotype compared to the *Abcd1* null mice despite higher VLCFA levels.^57^ In the opposed scenario, *Abcd1/VLCS* (very long‐chain acyl‐CoA synthetase) double KO mice present decreased levels of β‐oxidation of VLCFA; however, such reduction did not lead to increased VLCFA levels nor aggravated the phenotype of the *Abcd1* single‐mutant.^58^ In addition, further destabilization of myelin sheaths in *Abcd1/Mag* (myelin‐associated glycoprotein) double KO mice did not trigger inflammatory demyelination, which indicates that disruption of the Mag‐mediated glia‐axonal interactions is not sufficient to trigger the neuroinflammation seen in patients.^59^ Contrary to the mouse models described above, additional defects in plasmalogen synthesis in *Abcd1*/*Pex7* double KO mice recapitulate the most severe form of ALD, presenting inflammatory cerebral demyelination, together with axonal loss and reactive gliosis.^60^ The aforementioned different extra impairments on peroxisomal or related functions result, in some cases, in a more pronounced phenotype that ultimately highlight the importance of peroxisomes in several biological systems. Unfortunately, there are little, if any, follow up studies on these ALD mouse models.

**TABLE 2:** Comparison between the main findings of the double knockout mice and the ALD mouse

Genotype	Main findings in comparison to ALD mouse model
*Abcd1* ^*−/y*^ */Abcd2* ^*−/−*^	Higher VLCFA accumulation in the spinal cord and adrenal glands, accelerated motor defects and greater levels of oxidative damage in the spinal cord
*Abcd1* ^*−/y*^ */ELOVL1* ^*+/−*^	Higher VLCFA levels in brain and spinal cord
*Abcd1* ^*−/y*^ */Vlcs* ^*−/−*^	Decreased VLCFA β‐oxidation and similar levels of VLCFA
*Abcd1* ^*−/y*^ */Mag* ^*−/−*^	Increased myelin destabilization
*Abcd1* ^*−/y*^ */Pex7* ^*−/−*^	Impaired biosynthesis of plasmalogens, demyelination of CNS and PNS, increased axonal loss, and reactive gliosis

Peroxisomes have crucial roles in neural networks as supported by several metabolic disorders presenting clear disruptions in the nervous system.^61^ In agreement with this, deletion of functional peroxisomes from all neural cells in *Nes‐Pex5* ^*−/−*^ mice caused affected neuronal migration in development and axonal degeneration in adulthood.^62^ Further, impairment of peroxisomal function in neural‐ and astrocytic‐specific *Pex5* ^*−/−*^ mice did not produce any major impact on neurological functioning.^63^ Strikingly, no peroxisomal metabolism in oligodendrocytes in *CNPase‐Pex5* ^*−/−*^ mice resulted in axonopathy throughout the central nervous system, leading to axonal loss, demyelination and neuroinflammation in adult mice. This study revealed a fundamental role for oligodendrocytes in axonal pathology in ALD.^64^ Noteworthy, oligodendrocytes account for the majority of VLCFA synthesis and degradation of the brain.^57^, ^64^ Given that elevated VLCFA levels is the signature of virtually all neurodegenerative peroxisomal disorders, these findings suggest a prominent role for oligodendrocyte peroxisomal metabolism to support axons.^65^

## 8 CASE REPORT OF ALD IN A CHIMPANZEE

In 2017, a case report described an 11‐year‐old male chimpanzee with signs and symptoms resembling childhood cerebral ALD, including occasional erratic behavior, impaired vision, and difficulty swallowing.^66^ A brain MRI showed leukodystrophy with involvement of the frontal lobes, the genu of the corpus callosum and a relatively symmetric involvement of the bifrontal periventricular white matter. In addition, there was peripheral contrast enhancement which indicated active demyelinating lesions similar to childhood cerebral ALD. Compared to healthy chimpanzees, plasma VLCFA levels of this male were highly elevated. DNA analysis revealed a pathogenic missense mutation (p.Arg554His) that has also been reported in 50 ALD patients (https://adrenoleukodystrophy.info). At the time of diagnosis the mother of the chimpanzee was deceased already and there was no genomic DNA available to determine if this *ABCD1* mutation was inherited or de novo.

## 9 MODELING ALD WITH INDUCED PLURIPOTENT STEM CELLS

Recent developments in differentiation protocols for iPSCs enabled the generation of human cell types of different lineages. As consequence, iPSC‐derived cultures provide an in vitro platform to study disease‐relevant cell types that would otherwise not be accessible. In addition, the differentiated cells maintain the genetic background of the patient, which offers the opportunity to investigate complex genetic disorders. These properties make iPSCs attractive model systems with great potential to complement and expand our knowledge on ALD.

iPSC technology has begun to emerge in ALD research. At the pluripotent stem cell stage, iPSC from ALD patients do not accumulate VLCFA; however, a study reported elevated levels of VLCFA in early passage iPSC‐derived from childhood cerebral ALD patients.^6^, ^67^, ^68^ Interestingly, undifferentiated iPSCs from ALD patients showed dysregulated expression of genes involved in peroxisome abundance and neuroinflammation.^68^ In contrast to iPSCs, iPSC‐derived oligodendrocytes, astrocytes, and neurons derived from AMN and cerebral ALD patients showed elevated levels of VLCFA relative to healthy controls.^5^, ^6^ iPSC‐derived oligodendrocytes derived from AMN patients showed lower VLCFA levels than those derived from cerebral ALD patients.^5^, ^6^ In addition, generated oligodendrocytes and astrocytes presented higher saturated VLCFA levels, with the latter also showing higher expression of inflammatory markers and a greater response to cytokines.^5^ Despite the low number of cell lines included in these studies, these iPSC‐based findings are in line with a human study that demonstrated a correlation between C26:0 levels in normal‐appearing white matter and the clinical phenotype.^69^

The brain microvascular endothelial cells (BMECs) are a major cellular component of the brain‐blood barrier (BBB) responsible for its selective permeability.^70^ iPSCs from childhood cerebral ALD patients differentiated to BMECs presented decreased barrier integrity as seen by decreased trans‐endothelial electrical resistance. In addition, ALD BMECs accumulated more lipid droplets than normal BMECs.^71^ In addition, transcriptome analysis showed a decreased expression of genes involved in cell‐to‐cell attachment and an increased expression of genes related to inflammation. Although other BBB important cell types like pericytes and astrocytes are missing in this model, such leaky barrier would influence the development of cerebral ALD, characterized by immune cells infiltration in the CNS that accelerates the demyelination process.

New differentiation approaches to generate more advanced iPSC‐derived models are evolving rapidly. iPSCs from ALD patients have also been differentiated to microglia cells and in 3D to cerebral organoids, yet the focus of both studies was the generation of differentiation protocols and the patient‐derived produced cultures were not characterized.^72^, ^73^ Taken together, iPSC cultures have the potential to elucidate disease mechanisms by their ability to generate patient specific cell types that were previously inaccessible.

## 10 EVOLUTIONARY CONSERVED MECHANISMS IN PLANTS

The *Arabidopsis thaliana* homolog of *ABCD1*, named *At_ABCD1* (alternative names are: *CTS*, *PED3*, *PXA1, ACN2*), encodes a full‐size peroxisomal transporter of fatty acids.^74^, ^75^, ^76^ At_ABCD1 controls the shift between dormancy and germination by promoting seed growth, and it is involved in root development and fertility.^76^, ^77^, ^78^, ^79^, ^80^ Mutants lacking At_ABCD1 accumulate fatty acyl CoAs and present seedling germination deficiencies in the absence of sucrose.^74^, ^75^, ^76^ Interestingly, expression of human ABCD1 and ABCD2 proteins in *Arabidopsis thaliana* resulted in their correct peroxisomal localization, which is mediated by At_PEX19.^81^ Moreover, expression of human ABCD2 complemented the seed germination deficit of At_ABCD1 mutants, but expression of either ABCD1 or ABCD2 failed to rescue seedling establishment.^81^ Overall, these results support that the process of peroxisomal targeting of proteins is evolutionarily conserved, but suggest divergence in function and/or substrate specificity for ABCD transporters among plants and mammals.

## 11 CONCLUDING REMARKS

In this review, we have discussed the multiple models that have been generated to investigate ALD. This plethora of experimental models has advanced our understanding of the biochemical and cellular aspects underlying this complex disorder. However, despite extensive research, there has been no translation to the clinic and it is still not known why some patients develop the cerebral form of the disease or what is the role of VLCFA accumulation in the pathogenesis of ALD.

Our progress might be hampered by limitations of our current model systems. Even though some crucial components of the disease can be modeled, until now none of the model systems that have been developed can recapitulate ALD entirely. Specifically, accumulation of VLCFAs has been considered sufficient to label ABCD1‐deficient animals as valid models of ALD. However, animal models fail to develop cerebral white matter degeneration, the most severe and distinctive feature of the leukodystrophy. Still, each model develops different phenotypes, all related to a specific feature of ALD and associated with the same biochemical defect, which leaves open the opportunity to study multiple aspects of the disorder with the variety of model systems available.

ABCD1 deficiency impacts in one way or another the nervous system of the various research models of ALD. Although anecdotic, ABCD1 disruption resulted in cerebral ALD in chimpanzee, a nonhuman primate highly representing the clinical neurological phenotype and disease pathophysiology. Therefore, iPSC‐derived cultures, more specifically cerebral organoids, from cerebral ALD patients might provide the missing insights to further understand the neurobiology underlying ALD. The assortment of ALD model systems from uni‐ to multicellular organisms and from in vitro to in vivo approaches has provided, but will also yield new insights and directions for future studies to understand ALD.
